# Assessment of ORAI1-mediated basal calcium influx in mammary epithelial cells

**DOI:** 10.1186/1471-2121-14-57

**Published:** 2013-12-20

**Authors:** Diana GF Ross, Chanel E Smart, Iman Azimi, Sarah J Roberts-Thomson, Gregory R Monteith

**Affiliations:** 1 School of Pharmacy, The University of Queensland, Pharmacy Australia Centre of Excellence, 20 Cornwall St, Woolloongabba, QLD, Australia; 2 University of Queensland Centre for Clinical Research (UQCCR), Building 71/918 Royal Brisbane and Women’s Hospital, Herston, QLD 4029, Australia

## Abstract

**Background:**

The entry of calcium ions into mammary gland epithelial cells is one of the least well-understood processes in the transport of calcium into milk during lactation. The store-operated calcium entry channel ORAI1, has been suggested as a potential mechanism for the entry of Ca^2+^ into mammary gland epithelial cells from the maternal blood supply during lactation. The down regulation of the canonical ORAI1 activator STIM1 during lactation suggests that other known ORAI activators such as STIM2 and SPCA2 may be important during lactation.

**Results:**

Differentiation of HC11 mammary gland epithelial cells was associated with enhanced basal Ca^2+^ influx. Silencing of *Orai1* abolished this enhancement of Ca^2+^ influx. *Stim2* had a modest effect on Ca^2+^ influx in this *in vitro* model of lactation, whereas *Stim1* and *Spca2* silencing had no effect. Despite pronounced increases in *Spca2* mRNA during lactation there was no change in the generation of the alternative splice product generated by Mist1, which increases during lactation.

**Conclusions:**

These studies support the hypothesis that lactation is associated with a remodelling of Ca^2+^ influx and this is associated with enhancement of basal Ca^2+^ influx. This enhanced Ca^2+^ influx appears to occur through the calcium channel Orai1.

## Background

Lactation is the result of the finely orchestrated differentiation of mammary epithelial cells that gives them the ability to secrete milk. Mammary epithelial cells are unique in their ability to differentiate into lactogenic phenotypes and then dedifferentiate back to a quiescent form, in response to steroid and peptide hormones (reviewed by [1]). Milk provides an energy source, proteins and essential nutrients for the neonate, one of the key components of which is calcium (Ca^2+^). The rapid growth of the neonate, particularly the calcification of bones and teeth, places a high demand for Ca^2+^ in milk. Depending on the species, the concentration of total Ca^2+^ in milk ranges from 8 to 60 mM [2], a level well above the maternal blood level of total Ca^2+^. The secretory pathway and the apical plasma membrane play important roles in the transport of Ca^2+^ into milk [2-4].

Despite the importance of Ca^2+^ enrichment of milk, only recently have the Ca^2+^ transporters responsible for the accumulation of Ca^2+^ into milk begun to be identified. The best-characterized protein involved in the enrichment of milk with Ca^2+^ is the plasma membrane Ca^2+^ ATPase isoform 2 (PMCA2). This calcium efflux pump has a very restricted tissue expression and is present in specific parts of the brain and the inner ear [5-8]. PMCA2 is markedly up regulated during lactation, particularly splice variant PMCA2bw [5-7,9], which localizes to the apical membrane of secretory cells [10,11]. PMCA2 null mice show a 60% reduction in milk Ca^2+^ content, providing direct evidence for the role of PMCA2 in the apical transport of Ca^2+^ into milk during lactation [12].

The sequestration of Ca^2+^ into the secretory pathway during lactation appears to occur via the Golgi localized pump - secretory pathway Ca^2+^-ATPase isoform 2 (SPCA2). Like PMCA2, SPCA2 has a restricted tissue distribution [13] and is significantly up regulated during lactation [14]. SPCA2 may also have a dual role in lactation due to its Mn^2+^ pumping ability [15]. Both Ca^2+^ and Mn^2+^ are essential for enzymes necessary for the correct post-translational modification of milk proteins and lactose production [16].

Several different Ca^2+^ permeable ion channels are proposed as the mechanism by which Ca^2+^ enters the mammary epithelial cell from the maternal blood supply during lactation. Calcium channels suggested as involved in this pathway include TRPV5 and TRPV6 [17,18]. However, recent studies suggest that the Orai1 calcium channel may be responsible for calcium influx into the mammary epithelial cell during lactation, since *Orai1* mRNA levels increase in the mouse mammary gland during lactation [19]. Indeed, Orai1 is at the basolateral membrane in mammary epithelial cells [20]. ORAI1 is the canonical mechanism for store operated calcium entry (SOCE). SOCE is the activation of calcium influx into the cell upon the depletion of intracellular stores of Ca^2+^. Such a mechanism could be a powerful feedback loop to balance demand (the transport of Ca^2+^ into milk) with supply (the influx of Ca^2+^ into the mammary gland epithelial cell). Endoplasmic Ca^2+^ store level depletion is detected by STIM proteins; upon Endoplasmic Reticulum (ER) Ca^2+^ depletion, STIM proteins oligomerize and localize to ER-plasma membrane positions where they activate ORAI channels and promote SOCE [21-26]. The Orai1 isoform of ORAI channels is up regulated in mammary gland tissue samples taken from mice at lactation [19]. However, levels of the canonical Orai1 activator Stim1 decline during lactation. The related isoform Stim2 is suggested as the possible mechanism of activation of Orai1 during lactation as this isoform does not decrease during lactation and is linked to the regulation of basal Ca^2+^ influx [21]. Studies identifying the carboxyl terminal of SPCA2 as an activator of ORAI1 and the interaction between SPCA2 and ORAI1 in MCF-7 breast cancer cells, suggests that the up regulation of SPCA2 may not only serve to promote Ca^2+^ secretion during lactation, but also as an activator of Ca^2+^ influx. However, this has not been assessed in models of lactation. Another aspect of SPCA2 that has not yet been assessed in lactation is the role of the transcription factor MIST1, which is important in mammary gland development [27]. A novel, truncated form of Spca2 was identified in Mist1−/− mice, but the presence of this form of Spca2 and its potential role in the regulation of Ca^2+^ transport during lactation has not been assessed. In these studies we used the HC11 model to further explore Ca^2+^ influx in mammary gland epithelial cells and to define the potential role of MIST1 regulation of Spca2 splicing during mammary gland development.

## Results

### Basal Ca^2+^ influx is increased in differentiated HC11 cells

**Figure 1:**
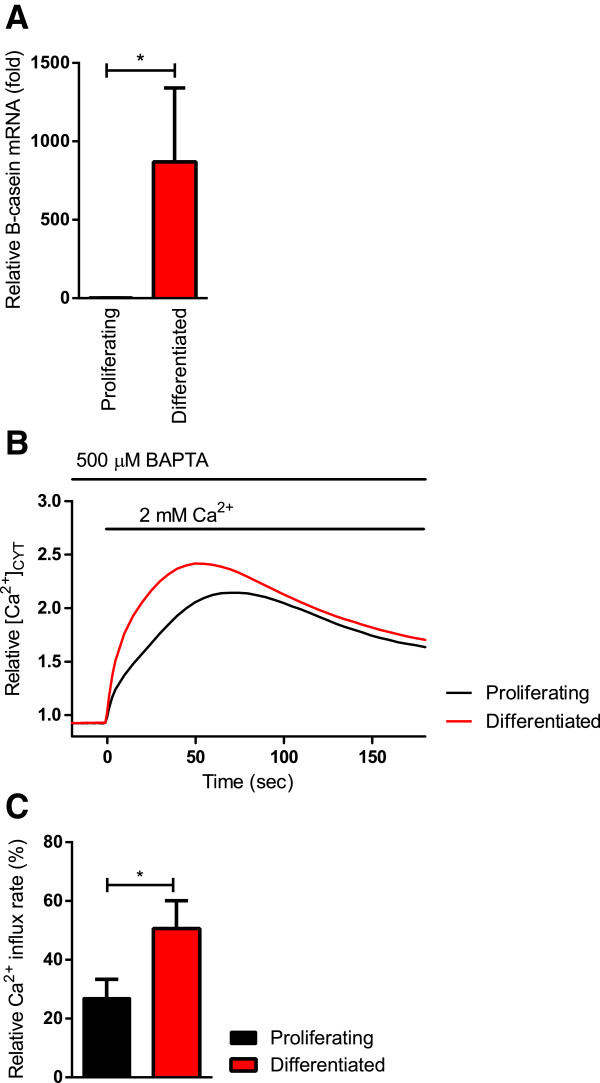
**HC11 cell differentiation and Ca**^**2+ **^**influx.** Total mRNA was isolated and assessed for an increase in *β-casein* mRNA to confirm differentiation using real-time RT-PCR. Calcium assays were conducted at the same time to measure changes in basal Ca^2+^ influx in response to differentiation. **A ***β-Casein* mRNA levels increased in differentiated HC11 cells in comparison to proliferating cells. Data were normalized to *Ppib* and *Actb* mRNA, and are shown as relative fold expression to differentiated HC11 cells (n = 4; mean ± SD, *P < 0.05). **B** Basal Ca^2+^ influx is shown as a trace of mean fluorescence relative to baseline in proliferating and differentiated HC11 cells. **C** Rate of basal Ca^2+^ influx, calculated as the slope between 0–5 s after 2 mM Ca^2+^ addition, in differentiated and proliferating HC11 cells. Rate is expressed as a percentage relative to differentiated cells treated with siNT and CPA (n = 4; mean ± SEM, *P < 0.05).

β-Casein is a known marker of lactogenesis in mammary epithelial cells necessary for the binding of calcium into micelles [2] and is a reliable marker in HC11 cells to show differentiation. HC11 cells were treated to induce differentiation to a lactogenic phenotype (as described in methods), which was assessed by the expression of *β-casein* at the mRNA level. Figure 1A demonstrates the increase *in β-casein* mRNA through real-time RT-PCR in differentiated cells in comparison to cells kept in maintenance media in a proliferative non-differentiated state.

Calcium assays were conducted to assess changes in basal Ca^2+^ influx in response to differentiation of HC11 cells. Basal Ca^2+^ influx (assessed by the rate of Ca^2+^ increase upon addition of extracellular Ca^2+^) significantly increased in differentiated cells (Figure 1B and C) indicating that an increase in basal Ca^2+^ influx accompanies β-casein induction and may be a characterizing feature of the changes associated with lactation. We then sought to determine the role of Orai1 in this enhancement of Ca^2+^ influx.

### Enhanced basal Ca^2+^ influx in differentiated HC11 cells is abolished by siRNA for Orai1

**Figure 2:**
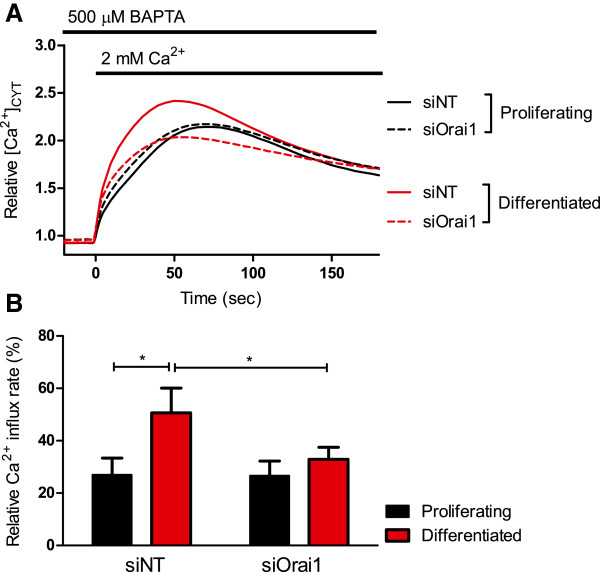
**Effect of Orai1 silencing on basal Ca**^**2+ **^**in HC11 cells.** Calcium assays were conducted to assess basal Ca^2+^ influx in proliferating and differentiated HC11 cells treated with siNT or siOrai1. **A** Basal Ca^2+^ influx is shown as a trace of mean fluorescence relative to baseline in proliferating and differentiated HC11 cells treated with siNT and siOrai1. **B** Rate of basal Ca^2+^ influx, calculated as the slope between 0–5 s after 2 mM Ca^2+^ addition, in differentiated and proliferating HC11 cells with siNT or siOrai1. Rate is expressed as a percentage relative to differentiated cells treated with siNT and CPA (n = 4; mean ± SEM, *P < 0.05).

The effect of siRNA to Orai1 (siOrai1) on basal Ca^2+^ influx in proliferative and differentiated HC11 cells was assessed. *Orai1* silencing eliminated the augmentation of basal Ca^2+^ influx associated with differentiation (Figure 2A and B) suggesting that Orai1 mediates this augmentation in differentiated HC11 cells.

**Figure 3:**
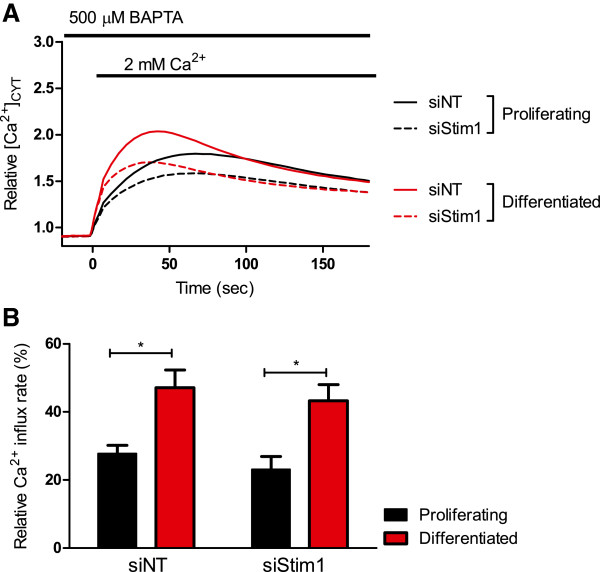
**Effect of Stim1 silencing on basal Ca**^**2+ **^**in HC11 cells.** Calcium assays were conducted to assess the basal Ca^2+^ influx in proliferating and differentiated HC11 cells treated with siNT or siStim1. **A** Basal Ca^2+^ influx is shown as a trace of mean fluorescence relative to baseline in proliferating and differentiated HC11 cells treated with siNT and siStim1. **B** Rate of basal Ca^2+^ influx, calculated as the slope between 0–5 s after 2 mM Ca^2+^ addition, in differentiated and proliferating HC11 cells with siNT or siStim1. Rate is expressed as a percentage relative to differentiated cells treated with siNT and CPA (n = 4; mean ± SEM, *P < 0.05).

**Figure 4:**
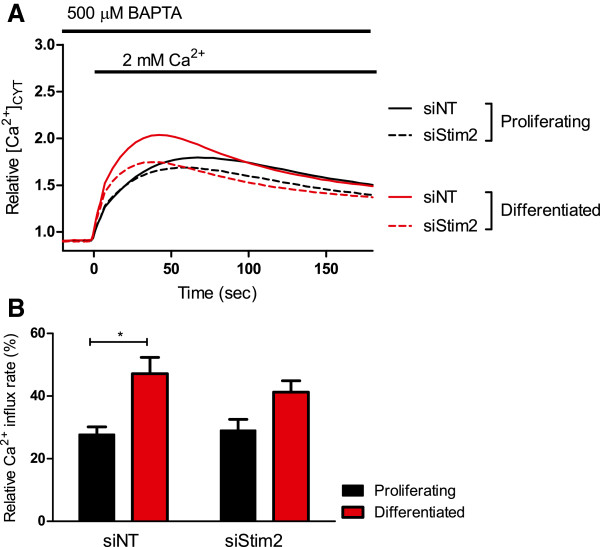
**Effect of Stim2 silencing on basal Ca**^**2+ **^**in HC11 cells.** Calcium assays were conducted to assess the basal Ca^2+^ influx in proliferating and differentiated HC11 cells treated with siNT or siStim2. **A** Basal Ca^2+^ influx is shown as a trace of mean fluorescence relative to baseline in proliferating and differentiated HC11 cells treated with siNT and siStim2. **B** Rate of basal Ca^2+^ influx, calculated as the slope between 0–5 s after 2 mM Ca^2+^ addition, in differentiated and proliferating HC11 cells with siNT or siStim2. Rate is expressed as a percentage relative to differentiated cells treated with siNT and CPA (n = 4; mean ± SEM, *P < 0.05).

*Stim1* silencing did not affect the augmented basal Ca^2+^ influx in differentiated HC11 cells (Figure 3A and B). Treatment of HC11 cells with siStim2 did not produce a significant inhibition of Ca^2+^ influx in differentiated cells (Figure 4A and B), however the significant increase in Ca^2+^ influx between differentiated and proliferating HC11 cells was abolished when cells were treated with siStim2 (Figure 4B). These results suggest a major role for Orai1 in Ca^2+^ influx in the HC11 model, with a modest role for Stim2.

### Spca2 in HC11 cells and mouse mammary tissue samples

**Figure 5:**
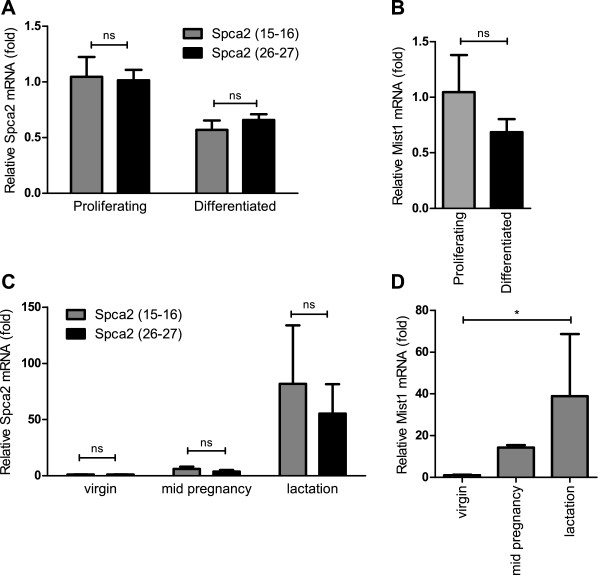
**Spca2 mRNA levels and Mist1-mediated truncated Spca2 in lactation.** Proliferating and differentiated HC11 cells were assessed for; **A**. *Spca2* mRNA spanning exons 15–16 and 26–27 and **B**. *Mist1* mRNA. Mouse mammary tissues isolated from virgin, mid pregnancy and lactation samples were assessed for; **C**. *Spca2* mRNA spanning exons 15–16 and 26–27 and **D**. *Mist1* mRNA*.* For all panels (n = 4; mean ± SD, ns denotes no significant difference *P* > 0.05).

**Figure 6:**
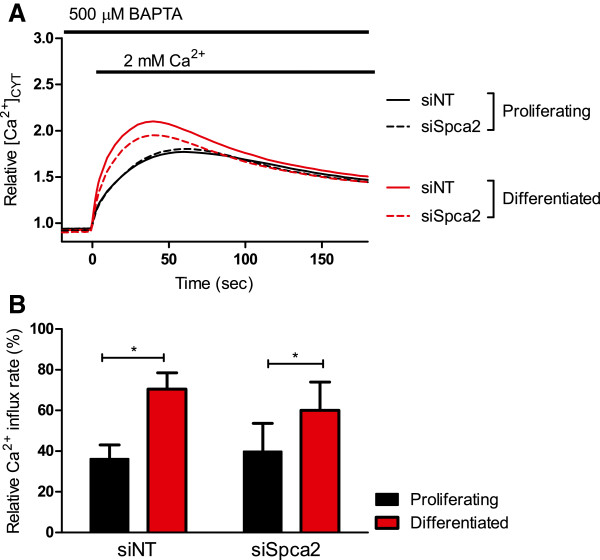
**Effect of Spca2 silencing on basal Ca**^**2+ **^**in HC11 cells.** Calcium assays were conducted to assess the basal Ca^2+^ influx in proliferating and differentiated HC11 cells treated with siNT or siSpca2. **A** Basal Ca^2+^ influx is shown as a trace of mean fluorescence relative to baseline in proliferating and differentiated HC11 cells treated with siNT and siSPCA2. **B** Rate of basal Ca^2+^ influx, calculated as the slope between 0–5 s after 2 mM Ca^2+^ addition, in differentiated and proliferating HC11 cells with siNT or siSpca2. Rate is expressed as a percentage relative to differentiated cells treated with siNT and CPA (n = 4; mean ± SD, *P < 0.05).

Truncated and full length *Spca2* transcripts [28] were assessed in proliferating and differentiated HC11 cells by comparing mRNA levels measured at exons 15–16 versus exons 26–27, (Figure 5A) and also in mouse mammary tissue samples isolated from virgin, mid pregnancy and lactation stages (Figure 5C). *Spca2* mRNA did not increase in the *in vitro* HC11 model and there was no change in the proportion of truncated versus full length transcripts (Figure 5A). However, *Spca2* mRNA did increase during lactation, although there was no significant difference between the different exon spanning regions of *Spca2* indicating that no truncated *Spca2* was present in any of the samples. We also assessed the levels of the transcription factor *Mist1*, which is speculated to regulate *Spca2* expression in pancreatic acinar cells [28], and found that *Mist1* levels did not significantly change in differentiated HC11 cells (Figure 5B). However, there was a trend of increased *Mist1* levels in mammary gland tissue samples from the lactating mice (Figure 5D) as previously described [27]. *Mist1* has been detected in differentiated SCp2 cells [27]. *Spca2* silencing had no significant effect on basal Ca^2+^ influx (Figure 6A and B) suggesting that in HC11 cells Spca2 does not play a role in Ca^2+^ influx regulation as suggested in other cell types [29].

### *β-Casein* levels are unchanged with *Orai1* silencing

**Figure 7:**
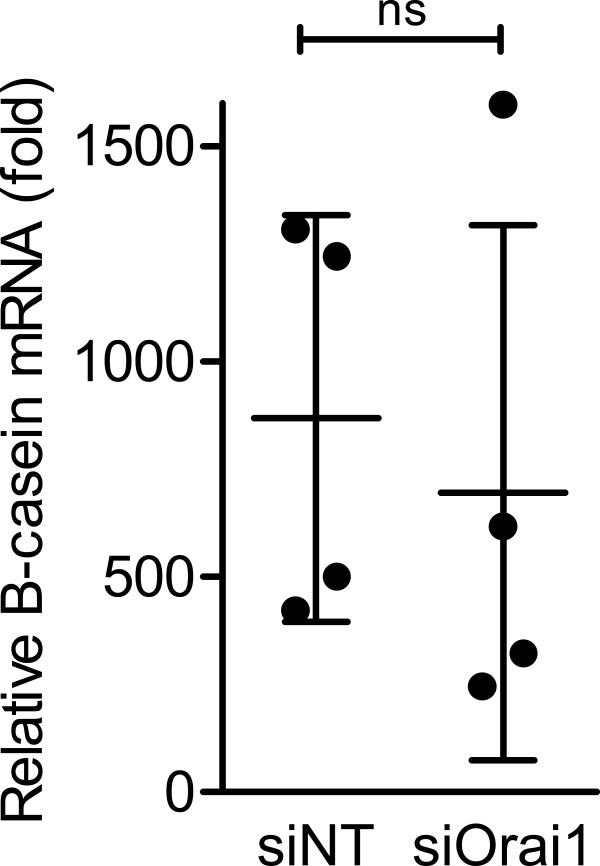
**Effect of Orai1 silencing on β-casein mRNA levels in HC11 cells.** β-Casein mRNA levels were assessed in differentiated HC11 cells treated with NT or Orai1 siRNA. Points on graph represent the average values from three wells across four independent experiments. Data were normalized to *Ppib* and *Actb* mRNA, and are shown as relative fold expression to differentiated HC11 cells. (n = 4; mean ± SD, *P < 0.05; ns denotes no significant difference).

To determine if the decrease in basal Ca^2+^ influx was an indirect result of siOrai1 inhibition of differentiation, *β-casein* mRNA levels were measured in siOrai1 treated differentiated HC11 cells. Real-time RT-PCR clearly showed that *β-casein* expression was not inhibited in siOrai1 treated cells (Figure 7). Therefore, although *Orai1* silencing abolishes the induction of increases in Ca^2+^ influx, it does not inhibit the induction of differentiation.

### *Orai1* mRNA levels do not change with differentiation

**Figure 8:**
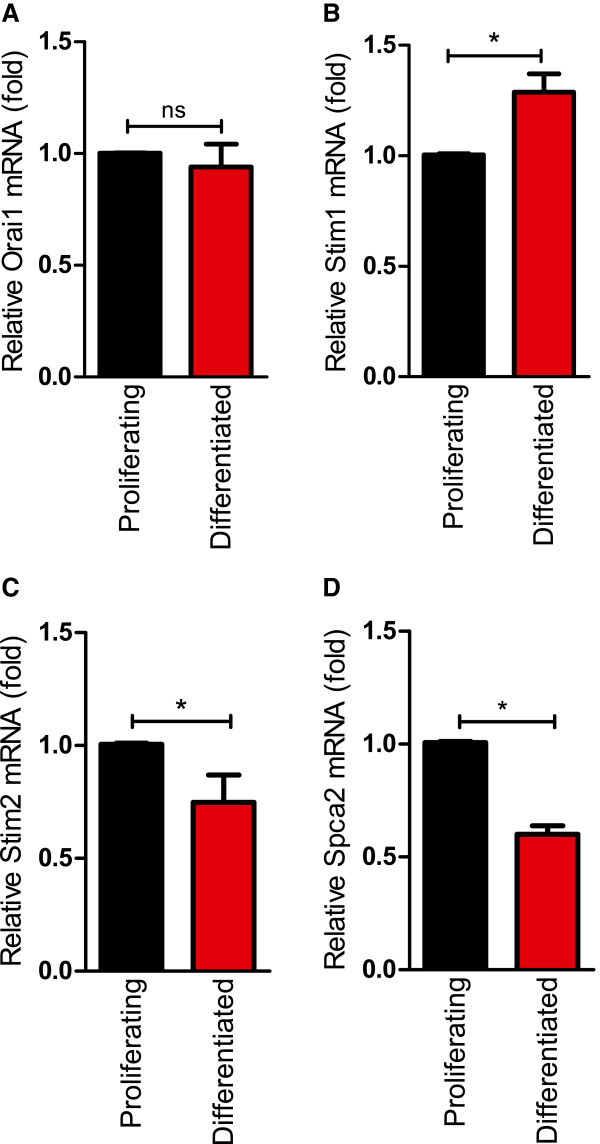
**Effect of differentiation in HC11 cells on mRNA expression of Orai1, Stim1, Stim2 and SPCA2.** HC11 cells were treated to induce differentiation or kept in maintenance media to keep them proliferating. Total mRNA was isolated 144 h post plating and assessed for *Orai1***(A)**, *Stim1***(B)**, *Stim2***(C)** and *Spca2***(D)** levels. Data were normalized to *Ppib* and *Actb* mRNA, and are shown as relative fold expression to differentiated HC11 cells. (n = 4; mean ± SD, *P < 0.05, ns denotes no significant difference).

Given the up regulation of Orai1-mediated Ca^2+^ influx associated with differentiation in HC11 cells, we assessed changes in the levels of *Orai1* and its known activators. *Orai1* (Figure 8A) mRNA did not significantly change in response to differentiation. However, differentiation was associated with a modest up regulation in *Stim1* mRNA (Figure 8B), and a modest down regulation of *Stim2* (Figure 8C) and *Spca2* (Figure 8D) mRNA levels.

## Discussion

The enrichment of milk with calcium is essential for the growing neonate. Our understanding of the specific calcium channels and pumps that are involved in this process is gradually evolving. However, the area that is least understood in the transport of Ca^2+^ into milk is the mechanism by which Ca^2+^ flows from the maternal blood supply into the mammary epithelial cell. *In vivo* studies implicate store operated Ca^2+^ entry as the calcium influx pathway involved, with *Orai1* mRNA levels increasing in mouse mammary glands during lactation [19]. Although *Orai1* silencing reduces Ca^2+^ influx in human breast cancer cell lines [19], no studies have directly assessed calcium influx mediated by this pathway in *in vitro* models of lactation. The possible roles of the Orai1 activators Stim1, Stim2 and Spca2 have also not been assessed through direct assessment of Ca^2+^ influx in mammary gland epithelial cells. These questions were addressed in this study.

HC11 cells are a mouse mammary epithelial cell line derived from COMMA-1D cells, isolated from mid-pregnant Balb/c mice mammary glands [30,31]. Unlike many other mammary cell models, HC11 cells do not need to be co-cultured with other cell types or with extracellular matrix proteins to help induce lactogenic differentiation [30,32-34], making it suitable for the calcium influx assays used in this study. The expression of β-casein is essential for Ca^2+^ accumulation into milk, as a significant amount of calcium is present in casein micelles [2,35]. Moreover, β-casein is a marker of differentiation in HC11 cells [36,37].

Differentiation of HC11 cells with lactogenic hormones resulted in a statistically significant increase in basal Ca^2+^ influx compared to undifferentiated controls. This result is consistent with the hypothesis that lactation is associated with the remodelling of mammary gland epithelial cells to a more Ca^2+^ permeable phenotype associated with elevated basal Ca^2+^ influx. This increase in Ca^2+^ influx is likely to be required to meet the demands of the secretion and efflux of Ca^2+^ from the mammary gland epithelial cell into milk.

Silencing of *Orai1* demonstrated that this augmented Ca^2+^ influx is via Orai1, since the difference in Ca^2+^ influx between differentiated and undifferentiated HC11 cells was abolished with the silencing of this calcium channel. Hence, consistent with elevated *Orai1* mRNA levels during lactation, Orai1 appears to be a major contributor of the enhanced basal Ca^2+^ influx in mammary gland epithelial cells from a lactating host. Orai1 along with its activator Stim2 are regulators of basal Ca^2+^ levels [21]. Our studies now suggest that this basal influx pathway is dynamic and may be up regulated during lactation. Future studies should assess how reported regulators of calcium transport during lactation, such as the calcium-sensing receptor, affect Orai1-mediated calcium influx in this and other models [38]).

McAndrews *et al.* compared 2 day (undifferentiated) and 8 day (with lactogenic hormones – differentiated) HC11 cultures and found elevated *Orai1* mRNA in HC11 cells at 8 days of differentiation [19]. These studies comparing cultures at day 8 with and without lactogenic hormones, suggest that it may be days in culture and/or confluence, which produces an increase in *Orai1* mRNA levels. Lactogenic hormones may be responsible for the enhancement of Orai1 basal activity in HC11 cells. Orai1 does not appear to be a key pathway in the differentiation of HC11 cells given that *Orai1* silencing had no effect on *β*-casein levels, this is in contrast to SCp2 cells where mammosphere formation was abolished with shOrai1 treatment [20]. The lack of effects of *Orai1* silencing on *β*-casein levels in HC11 cells indicates that the decrease in basal Ca^2+^ influx observed when *Orai1* was silenced was not simply a consequence of the inhibition of HC11 differentiation.

ORAI1 is activated via a number of different mechanisms including the canonical ORAI1 activator and calcium store sensor STIM1, its related isoform STIM2, and SPCA2. STIM2 is proposed as an ORAI1 activator in lactation due to its important role in basal influx in HeLa, HUVEC and HEK293T cells [21]. The maintenance of *Stim2* mRNA levels during lactation [19] and reduction in basal [Ca^2+^]_CYT_ in differentiated HC11 cells with *Stim2* silencing also support this role. Other mechanisms of ORAI1 activation during lactation have also been proposed, such as calcium store independent activation by SPCA2 [29]. Specific domains of SPCA2 protein activate ORAI1 [29], and *Spca2* mRNA levels are pronouncedly increased during lactation [14]. Activation of ORAI1 by SPCA2 may allow the demand for Ca^2+^ sequestration via this secretory pathway Ca^2+^ pump to promote the supply of Ca^2+^ through augmentation of Ca^2+^ influx. However, in these studies, and in contrast to *Orai1* silencing, neither *Stim1* nor *Spca2* silencing abolished the augmentation of Ca^2+^ influx induced by differentiation of HC11 mammary epithelial cells. It could be that some *in vitro* systems have calcium influx pathways that are more sensitive to spca2 dependent modulation of Orai1, such has recently been reported in Scp2 cells [20]. The ability of Stim2 silencing to abolish the enhanced Ca^2+^ influx associated with differentiation of HC11 cells, supports previous suggestions that Stim2 is a modulator of Orai1 during lactation, however, the inability of *Stim2* silencing to replicate the magnitude of *Orai1* silencing suggest other Orai1 activation mechanisms. Compensatory mechanisms amongst the Orai1 activators Stim1, Stim2 and Spca2 in this model and/or other factors such as the absence of coordinated polarization of mammary gland epithelial cell may be responsible for the results reported here. Further studies using 3D culture models in multiple mammary cell lines models, such as those recently published by Cross *et al.* in Spc2 cells [20], and *in vivo* studies with knockout animals are required to ultimately define the specific roles of Stim1, Stim2 and Spca2 and the activation of Orai1 in lactation.

## Conclusions

The influx of Ca^2+^ across mammary gland epithelial cells is a key step in the supply of Ca^2+^ to the growing neonate during lactation. These studies using HC11 mammary gland epithelial cells are consistent with recent *in vivo* studies of mRNA levels during mammary gland development suggesting that Orai1 is an important pathway of Ca^2+^ influx during lactation.

## Methods

### Cell culture

Mouse mammary epithelial cells (HC11) were propagated in maintenance media containing Roswell Park Memorial Institute (RPMI)-1640 Medium (R8757, Sigma Aldrich) supplemented with 10% fetal bovine serum (FBS) and 10 mg/mL bovine insulin, as previously described [19]. Cells were maintained in a 37°C, humidified 5% CO_2_, 95% air incubator. Cell lines were routinely tested for mycoplasma contamination.

### HC11 cell differentiation and cell culture treatments

HC11 cells were seeded at 3500 cells/well into 96 well plates in maintenance media containing 10 ng/mL murine EGF. Fresh maintenance media with EGF was added at 24 h and siRNA treatment performed at 48 h in the presence of EGF and insulin. Following 24 h siRNA treatment media was replaced with maintenance media for 24 h. After 48 h siRNA treatment, media was replaced with either maintenance media supplemented with 1 μM dexamethasone and 5 μg/mL ovine prolactin (L6520, Sigma Aldrich) to differentiate the cells, or with maintenance media for proliferating non-differentiated cells. Fresh maintenance media was added at 24 h with or without dexamethasone and prolactin, as appropriate. Calcium assays were performed 144 h post plating. RNA was isolated from cells after calcium measurements were taken. Experiments were conducted in triplicate wells and all experiments were performed independently on four occasions.

### siRNA transient transfection

siRNA transfection was performed using Dharmacon ON-TARGET*plus* SMARTpool™ siRNA (100 nM), comprising a pool of four siRNA sequences rationally designed with dual strand modification and use of an algorithm to reduce seed region matches. DharmaFECT1 transfection reagent (catalogue number T-2001-01) was used (0.4 μL/well) as per the manufacturer’s instructions. The following Dharmacon On-TARGETplus SMARTpool™ mouse siRNAs were used in this study: non-targeting (D-001810-10-05), *Orai1* (L-056431-02-005), *Stim1* (L-062376-00-0005), *Stim2* (L-055069-01-0005) and *Spca2* (L-065820-01-005). siRNA knockdown was checked using real-time RT-PCR for each experimental plate 24 h post treatment (Additional file 1).

### Calcium assays

Calcium assays were performed using a fluorometric imaging plate reader (FLIPR^TETRA^, Molecular Devices Corporation) using the no-wash cytosolic free calcium PBX Ca^2+^ Assay Kit (BD Biosciences) as previously described [39]. Cells were seeded in 96 well black-walled plates (Corning). Intracellular Ca^2+^ measurements were performed with an excitation intensity of 470–495 nm and a 515–575 nm emission filter. Fluorescent values were normalized to the starting fluorescence and were expressed as relative [Ca^2+^]_CYT_. The slope of the curve was calculated based on points at 0 to 5 s post Ca^2+^ addition and were expressed as a percentage relative to the average readings for differentiated cells treated with non-targeting siRNA with maximum Orai1-mediated Ca^2+^ influx induced by 10 μM cyclopiazonic acid (CPA).

### Real-time RT-PCR

Total RNA was isolated using the RNeasy Plus mini kit (Qiagen) as per the manufacturer’s instructions, and mRNA was quantitated using real time RT-PCR and a 7500 real time PCR system (Applied Biosystems). Mouse mammary tissues were obtained as previously described [19]. Mouse *β-casein* (Mm00839664_m1), *Orai1* (Mm00774349_m1), *Spca2* (Mm01242899_m1), *Spca2* exon 15–16 (Mm01242904_m1), *Spca2* exon 26–27 (Mm01242916_m1), *Mist1* (Mm00487695_m1), *Stim1* (Mm00486423_m1), and *Stim2* (Mm01223102_m1) were amplified using the TaqMan® gene expression assays, and the data normalized to *Ppib* (Mm00478295_m1) and *Actb* (Mm01205647_g1). Data were analyzed using the comparative C_t_ method as described previously [40]. All data were normalized to proliferating HC11 cells treated with non-targeting siRNA. Data are shown as mean plus SD (n = 4).

### Statistical analysis

All experimental treatments were conducted as three wells per experimental plate in quadruplicate. Results for the slope are presented as standard error of the mean (SEM) and statistical comparisons were performed using a two-way RM ANOVA matching by rows with a Bonferroni multiple comparison post-test. Real-time RT-PCR results are presented as standard deviation (SD) and were analyzed for significance using a paired t-test. All statistical analyzes were conducted using Prism Graph Pad, Version 5.04, Berkeley, CA, and significance was demonstrated at P < 0.05 where appropriate.

## Competing interests

The authors declare that they have no competing interests.

## Authors’ contributions

DR helped design and performed experiments and data analysis and wrote the paper. CS contributed to the writing of the paper and preparation of lactation samples. IA contributed to the writing and revision of the paper. SRT designed experiments and contributed to the data analysis and writing of the paper. GM designed experiments, and contributed to analysis of the data and wrote the paper. All authors read and approved the final manuscript.

## Supplementary Material

Additional file 1 **HC11 cells were treated with siRNA for *Orai1 * (A.), *Stim1 *(B.), *Stim2 *(C.) and *Spca2 *(D.).** Total mRNA was isolated 24 h after siRNA treatment before real-time. RT-PCR for assessment of gene silencing. Data were normalised to *Ppib* and *ActB* mRNA, and are shown as relative fold expression to siNT treated HC11 cells (n=4; mean +/- SD, *P < 0.05). Click here for file

## References

[B1] Borellini F Oka T Growth control and differentiation in mammary epithelial cells Environ Health Perspect 1989 14 85 99 2647487 10.1289/ehp.898085 PMC1567615

[B2] Neville MC Calcium secretion into milk J Mammary Gland Biol 2005 14 119 128 10.1007/s10911-005-5395-z 16025219

[B3] Shennan DB Peaker M Transport of milk constituents by the mammary gland Physiol Rev 2000 14 925 951 10893427 10.1152/physrev.2000.80.3.925

[B4] McManaman JL Neville MC Mammary physiology and milk secretion Adv Drug Deliv Rev 2003 14 629 641 10.1016/S0169-409X(03)00033-4 12706546

[B5] Reinhardt TA Filoteo AG Penniston JT Horst RL Ca2 + −ATPase protein expression in mammary tissue Am J Physiol-Cell Ph 2000 14 C1595 C1602 10.1152/ajpcell.2000.279.5.C1595 11029307

[B6] Reinhardt TA Horst RL Ca2 + −ATPases and their expression in the mammary gland of pregnant and lactating rats Am J Physiol 1999 14 C796 C802 10199809 10.1152/ajpcell.1999.276.4.C796

[B7] Strehler EE Treiman M Calcium pumps of plasma membrane and cell interior Curr Mol Med 2004 14 323 335 10.2174/1566524043360735 15101689

[B8] Grover AK Khan I Calcium pump isoforms: diversity, selectivity and plasticity Review article. Cell Calcium 1992 14 9 17 10.1016/0143-4160(92)90025-N 1311640

[B9] VanHouten JN Neville MC Wysolmerski JJ The calcium-sensing receptor regulates plasma membrane calcium adenosine triphosphatase isoform 2 activity in mammary epithelial cells: a mechanism for calcium-regulated calcium transport into milk Endocrinology 2007 14 5943 5954 10.1210/en.2007-0850 17823248 PMC7108505

[B10] Chicka MC Strehler EE Alternative splicing of the first intracellular loop of plasma membrane Ca2 + −ATPase isoform 2 alters its membrane targeting J Biol Chem 2003 14 18464 18470 10.1074/jbc.M301482200 12624087

[B11] Grati M Aggarwal N Strehler EE Wenthold RJ Molecular determinants for differential membrane trafficking of PMCA1 and PMCA2 in mammalian hair cells J Cell Sci 2006 14 2995 3007 10.1242/jcs.03030 16803870

[B12] Reinhardt TA Lippolis JD Shull GE Horst RL Null mutation in the gene encoding plasma membrane Ca2 + −ATPase isoform 2 impairs calcium transport into milk J Biol Chem 2004 14 42369 42373 10.1074/jbc.M407788200 15302868

[B13] Xiang M Mohamalawari D Rao R A novel isoform of the secretory pathway Ca2+, Mn(2+)-ATPase, hSPCA2, has unusual properties and is expressed in the brain J Biol Chem 2005 14 11608 11614 10.1074/jbc.M413116200 15677451

[B14] Faddy HM Smart CE Xu R Lee GY Kenny PA Feng M Rao R Brown MA Bissell MJ Roberts-Thomson SJ Monteith GR Localization of plasma membrane and secretory calcium pumps in the mammary gland Biochem Biophys Res Commun 2008 14 977 981 10.1016/j.bbrc.2008.03.003 18334228 PMC3234104

[B15] Vanoevelen J Dode L Van Baelen K Fairclough RJ Missiaen L Raeymaekers L Wuytack F The secretory pathway Ca2+/Mn2 + −ATPase 2 is a Golgi-localized pump with high affinity for Ca2+ ions J Biol Chem 2005 14 22800 22808 10.1074/jbc.M501026200 15831496

[B16] Lee WJ Monteith GR Roberts-Thomson SJ Calcium transport and signaling in the mammary gland: targets for breast cancer Biochim Biophys Acta 2006 14 235 255 16410040 10.1016/j.bbcan.2005.12.001

[B17] VanHouten JN Wysolmerski JJ Transcellular calcium transport in mammary epithelial cells J Mammary Gland Biol 2007 14 223 235 10.1007/s10911-007-9057-1 17999165

[B18] Hoenderop JGJ Nilius B Bindels RJM Epithelial calcium channels: from identification to function and regulation Pflug Arch Eur J Phy 2003 14 304 308 10.1007/s00424-003-1045-8 12684797

[B19] McAndrew D Grice DM Peters AA Davis FM Stewart T Rice M Smart CE Brown MA Kenny PA Roberts-Thomson SJ Monteith GR ORAI1-mediated calcium influx in lactation and in breast cancer Mol Cancer Ther 2011 14 448 460 10.1158/1535-7163.MCT-10-0923 21224390

[B20] Cross BM Hack A Reinhardt TA Rao R SPCA2 Regulates Orai1 Trafficking and Store Independent Ca(2+) Entry in a Model of Lactation PLoS One 2013 14 e67348 10.1371/journal.pone.0067348 23840669 PMC3696057

[B21] Brandman O Liou J Park WS Meyer T STIM2 is a feedback regulator that stabilizes basal cytosolic and endoplasmic reticulum Ca2+ levels Cell 2007 14 1327 1339 10.1016/j.cell.2007.11.039 18160041 PMC2680164

[B22] Liou J Fivaz M Inoue T Meyer T Live-cell imaging reveals sequential oligomerization and local plasma membrane targeting of stromal interaction molecule 1 after Ca2+ store depletion Proc Natl Acad Sci USA 2007 14 9301 9306 10.1073/pnas.0702866104 17517596 PMC1890489

[B23] Liou J Kim ML Heo WD Jones JT Myers JW Ferrell JE Jr Meyer T STIM is a Ca2+ sensor essential for Ca2 + −store-depletion-triggered Ca2+ influx Curr Biol 2005 14 1235 1241 10.1016/j.cub.2005.05.055 16005298 PMC3186072

[B24] Wu MM Buchanan J Luik RM Lewis RS Ca2+ store depletion causes STIM1 to accumulate in ER regions closely associated with the plasma membrane J Cell Biol 2006 14 803 813 10.1083/jcb.200604014 16966422 PMC2064335

[B25] Xu P Lu J Li Z Yu X Chen L Xu T Aggregation of STIM1 underneath the plasma membrane induces clustering of Orai1 Biochem Biophys Res Commun 2006 14 969 976 10.1016/j.bbrc.2006.09.134 17045966

[B26] Luik RM Wu MM Buchanan J Lewis RS The elementary unit of store-operated Ca2+ entry: local activation of CRAC channels by STIM1 at ER-plasma membrane junctions J Cell Biol 2006 14 815 825 10.1083/jcb.200604015 16966423 PMC2064336

[B27] Zhao Y Johansson C Tran T Bettencourt R Itahana Y Desprez P-Y Konieczny SF Identification of a basic helix-loop-helix transcription factor expressed in mammary gland alveolar cells and required for maintenance of the differentiated state Mol Endocrinol 2006 14 2187 2198 10.1210/me.2005-0214 16645041

[B28] Garside VC Kowalik AS Johnson CL DiRenzo D Konieczny SF Pin CL MIST1 regulates the pancreatic acinar cell expression of Atp2c2, the gene encoding secretory pathway calcium ATPase 2 Exp Cell Res 2010 14 2859 2870 10.1016/j.yexcr.2010.06.014 20599950 PMC3342848

[B29] Feng M Grice DM Faddy HM Nguyen N Leitch S Wang Y Muend S Kenny PA Sukumar S Roberts-Thomson SJ Store-independent activation of Orai1 by SPCA2 in mammary tumors Cell 2010 14 84 98 10.1016/j.cell.2010.08.040 20887894 PMC2950964

[B30] Ball RK Friis RR Schoenenberger CA Doppler W Groner B Prolactin regulation of beta-casein gene expression and of a cytosolic 120-kd protein in a cloned mouse mammary epithelial cell line EMBO J 1988 14 2089 2095 3416834 10.1002/j.1460-2075.1988.tb03048.x PMC454494

[B31] Danielson KG Oborn CJ Durban EM Butel JS Medina D Epithelial mouse mammary cell line exhibiting normal morphogenesis in vivo and functional differentiation in vitro Proc Natl Acad Sci USA 1984 14 3756 3760 10.1073/pnas.81.12.3756 6587390 PMC345298

[B32] Streuli CH Bailey N Bissell MJ Control of mammary epithelial differentiation: basement membrane induces tissue-specific gene expression in the absence of cell-cell interaction and morphological polarity J Cell Biol 1991 14 1383 1395 10.1083/jcb.115.5.1383 1955479 PMC2289247

[B33] Lin CQ Bissell MJ Multi-faceted regulation of cell differentiation by extracellular matrix FASEB J 1993 14 737 743 8330681 10.1096/fasebj.7.9.8330681

[B34] Chammas R Taverna D Cella N Santos C Hynes NE Laminin and tenascin assembly and expression regulate HC11 mouse mammary cell differentiation J Cell Sci 1994 14 Pt 4 1031 1040 7520040 10.1242/jcs.107.4.1031

[B35] Neville MC Watters CD Secretion of calcium into milk: review J Dairy Sci 1983 14 371 380 10.3168/jds.S0022-0302(83)81802-5 6302145

[B36] Taverna D Groner B Hynes NE Epidermal growth factor receptor, platelet-derived growth factor receptor, and c-erbB-2 receptor activation all promote growth but have distinctive effects upon mouse mammary epithelial cell differentiation Cell Growth Differ 1991 14 145 154 1676295

[B37] Venesio T Taverna D Hynes NE Deed R MacAllan D Ciardiello F Valverius EM Salomon DS Callahan R Merlo G The int-2 gene product acts as a growth factor and substitutes for basic fibroblast growth factor in promoting the differentiation of a normal mouse mammary epithelial cell line Cell Growth Differ 1992 14 63 71 1376141

[B38] Mamillapalli R VanHouten J Dann P Bikle D Chang W Brown E Wysolmerski J Mammary-specific ablation of the calcium-sensing receptor during lactation alters maternal calcium metabolism, milk calcium transport, and neonatal calcium accrual Endocrinology 2013 14 3031 3042 10.1210/en.2012-2195 23782944 PMC3749485

[B39] Grice DM Vetter I Faddy HM Kenny PA Roberts-Thomson SJ Monteith GR Golgi calcium pump secretory pathway calcium ATPase 1 (SPCA1) is a key regulator of insulin-like growth factor receptor (IGF1R) processing in the basal-like breast cancer cell line MDA-MB-231 J Biol Chem 2010 14 37458 37466 10.1074/jbc.M110.163329 20837466 PMC2988351

[B40] Aung CS Ye W Plowman G Peters AA Monteith GR Roberts-Thomson SJ Plasma membrane calcium ATPase 4 and the remodeling of calcium homeostasis in human colon cancer cells Carcinogenesis 2009 14 1962 1969 10.1093/carcin/bgp223 19755660

