# Supplementary material for: Assessment of ORAI1-mediated basal calcium influx in mammary epithelial cells
Source: BMC Cell Biol. 2013 Dec 20;14:57. doi: 10.1186/1471-2121-14-57 (PMC3878224; doi:10.1186/1471-2121-14-57)
Supplement: Additional file 1 — HC11 cells were treated with siRNA for Orai1 (A.), Stim1 (B.), Stim2 (C.) and Spca2 (D.). Total mRNA was isolated 24 h after siRNA treatment before real-time. RT-PCR for assessment of gene silencing. Data were normalised to Ppib and ActB mRNA, and are shown as relative fold expression to siNT treated HC11 cells (n=4; mean +/- SD, *P < 0.05). [file 1471-2121-14-57-S1.pdf]

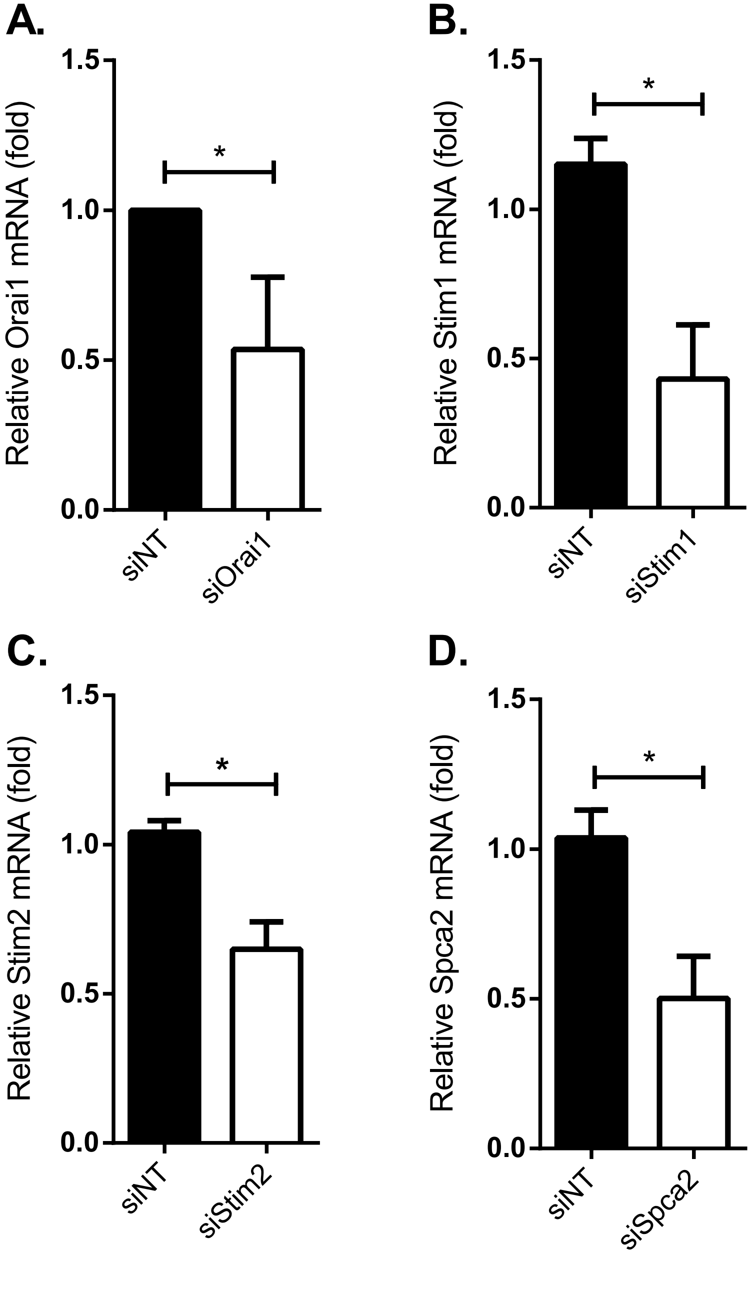

Additional file 1

HC11 cells were treated with siRNA for *Orai1* (A.), *Stim1* (B.), *Stim2* (C.) and *Spca2* (D.). Total mRNA was isolated 24 h after siRNA treatment before real-time RT-PCR for assessment of gene silencing. Data were normalised to *Ppib* and *ActB* mRNA, and are shown as relative fold expression to siNT treated HC11 cells.(n=4; mean +/- SD, **P* < 0.05)
